# Biomechanical impact of labiolingual diameter on endodontically treated anterior teeth with crown restoration under occlusal loading

**DOI:** 10.1590/1678-7757-2023-0439

**Published:** 2024-06-14

**Authors:** Miao LIANG, Lamu ZEYONG, Yongheng LI, Qiang CHEN, Xiangfeng MENG

**Affiliations:** 1 Nanjing University Affiliated Hospital of Medical School Nanjing Stomatological Hospital Nanjing China Nanjing University, Affiliated Hospital of Medical School, Nanjing Stomatological Hospital, Department of General Dentistry, Nanjing, 210008, China.; 2 Nanjing University Affiliated Hospital of Medical School Nanjing Stomatological Hospital Nanjing China Nanjing University, Affiliated Hospital of Medical School, Nanjing Stomatological Hospital, Department of Prosthodontics, Nanjing, 210008, China.; 3 Southeast University School of Biological Science & Medical Engineering Biomechanics Laboratory Nanjing China Southeast University, School of Biological Science & Medical Engineering, Biomechanics Laboratory, Nanjing, 210008, China.; 4 Nanjing University Affiliated Hospital of Medical School Nanjing Stomatological Hospital Nanjing China Nanjing University, Affiliated Hospital of Medical School, Nanjing Stomatological Hospital, Department of Prosthodontic Technology, Nanjing, 210008, China.

**Keywords:** Endodontically treated anterior tooth, Labiolingual diameter, Construction, Post and core technique, Finite element analysis

## Abstract

**Objective:**

To evaluate the effect of the labiolingual diameter and construction of an endodontically treated (ET) anterior tooth with crown restoration on stress distribution and biomechanical safety under occlusal loading.

**Methodology:**

Three-dimensional finite element models were generated for maxillary central incisors with all-ceramic crown restorations. The labiolingual diameters of the tooth, defined as the horizontal distance between the protrusion of the labial and lingual surfaces, were changed as follows: (D1) 6.85 mm, (D2) 6.35 mm, and (D3) 5.85 mm. The model was constructed as follows: (S0) vital pulp tooth; (S1) ET tooth; (S2) ET tooth with a 2 mm ferrule, restored with a fiber post and composite resin core; (S3) ET tooth without a ferrule, restored with a fiber post and composite resin core. A total of 12 models were developed. In total, two force loads (100 N) were applied to the crown’s incisal edge and palatal surface at a 45° oblique angle to the longitudinal axis of the teeth. The Von Mises stress distribution and maximum stress of the models were analyzed.

**Results:**

Regardless of the loading location, stress concentration and maximum stress (34.07~66.78MPa) in all models occurred in the labial cervical 1/3 of each root. Both labiolingual diameter and construction influenced the maximum stress of the residual tooth tissue, with the impact of the labiolingual diameter being greater. A reduction in labiolingual diameter led to increased maximum stress throughout the tooth. The ferrule reduced the maximum stress of the core of S2 models (7.15~10.69 MPa), which is lower compared with that of S3 models (19.45~43.67 MPa).

**Conclusion:**

The labiolingual diameter exerts a greater impact on the biomechanical characteristics of ET anterior teeth with crown restoration, surpassing the influence of the construction. The ferrule can reduce the maximum stress of the core and maintain the uniformity of stress distribution.

## Introduction

Full crown restorations are widely accepted in restorative dentistry, providing enhanced aesthetics, function, and pronunciation, in which significant crown tissue loss occurs on the incisor due to factors such as trauma or caries.^1,2^ The quantity of remaining coronal tooth tissue significantly influences the type of final restoration, especially for endodontically treated (ET) teeth.^3,4^ A few studies indicate that a post is not necessary for an ET anterior tooth with minimal loss of tooth structure.^1,5,6^ However, if significant tooth structure is lost, and an anterior tooth requires crown preparation after endodontic treatment, a post may be necessary to retain the core and ensure resistance to functional forces.^5,7^ Fiber post and composite resin cores, valued for their mechanical properties and aesthetics, find extensive applications in clinical practice.^6,8,9^ Moreover, the presence of ferrule positively impacts the fracture resistance of ET teeth, with a more successful prognosis anticipated if healthy dentin extending 1.5–2 mm coronal to the crown margin is circumferentially available.^6,7,10,11^

Although existing literature has reached a consensus on the construction of ET teeth, a lack of information remains on the labiolingual diameter of the ET incisor. In clinical practice, alterations in the labiolingual diameter of the ET incisor can affect the effectiveness of crown restoration. Tooth preparation reduces residual coronal tooth tissue; in these cases, excessive preparation may be conducted to achieve a sufficient ceramic layer thickness for better aesthetics, further reducing the residual tooth tissue.^12,13^ Additionally, variation in tooth size between ethnicities contributes to differences, with Asians and Europeans having smaller intact maxillary central incisors than Oceanians and Africans.^14,15^ This results in a reduced labiolingual diameter of ET teeth. Individuals with chronic gastric regurgitation may experience acid erosion or abrasion on the lingual surfaces of their incisors, leading to further reduction of labiolingual diameters.^16^ However, the current literature is limited in addressing the specific effects of reduced labiolingual diameter on the biomechanics of ET anterior teeth.

Thus, this study aimed to evaluate the impact of labiolingual diameter and construction of an ET maxillary central incisor with crown restoration on stress distribution and biomechanical safety under simulated occlusal loading using the three-dimensional finite element method (FEM).

## Methodology

In this section, numerical simulations were conducted using the finite element software ABAQUS 6.12 (Dassault Simulia, USA) to gain insight into the mechanical behavior of ET anterior teeth. Considering three labiolingual diameters and four constructions, 12 FEM models were generated. In total, two functional occlusal states were simulated and the maximum Von Mises stress and stress distribution of the tooth models (dentin, post, and core) were investigated.

### Generation of 12 FEM models

**Figure 1:**
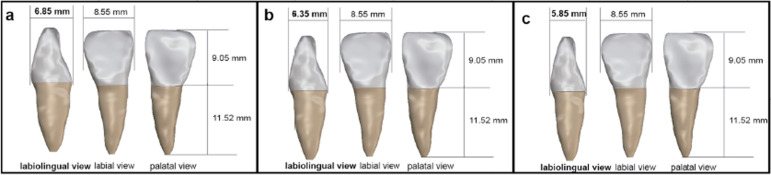
Tooth models with three different labiolingual diameters: (a) D1 model, (b) D2 model, and (c) D3 model. The labiolingual diameters of the tooth model were the horizontal distance between the protrusion of the labial and lingual surfaces, as shown in the labiolingual view.

A sound maxillary central incisor was scanned using Cone beam Computer Tomography (CBCT, NEWTOM, Italy), following teeth size and shape standards detailed by Wheeler.^17^ Using Mimics 19.0 software (Materialise, Belgium), solid models of the central incisor, segmented into enamel, dentin, and pulp, were generated based on CBCT image data. The tooth model, used as the reference group (D1 model), was further optimized after smoothing and denoising. Subsequently, the D1 model was modified by narrowing in the labiolingual direction, being 0.5 mm for D2 and 1.0 mm for D3, using Rhino 6.0 software (Robert McNeel, America) to obtain their models. Figure 1 illustrates the specific sizes of the three models. To simulate the alveolar bone, a 0.2 mm-thick periodontal ligament supported the tooth, with the root embedded in a cylinder.

**Figure 2:**
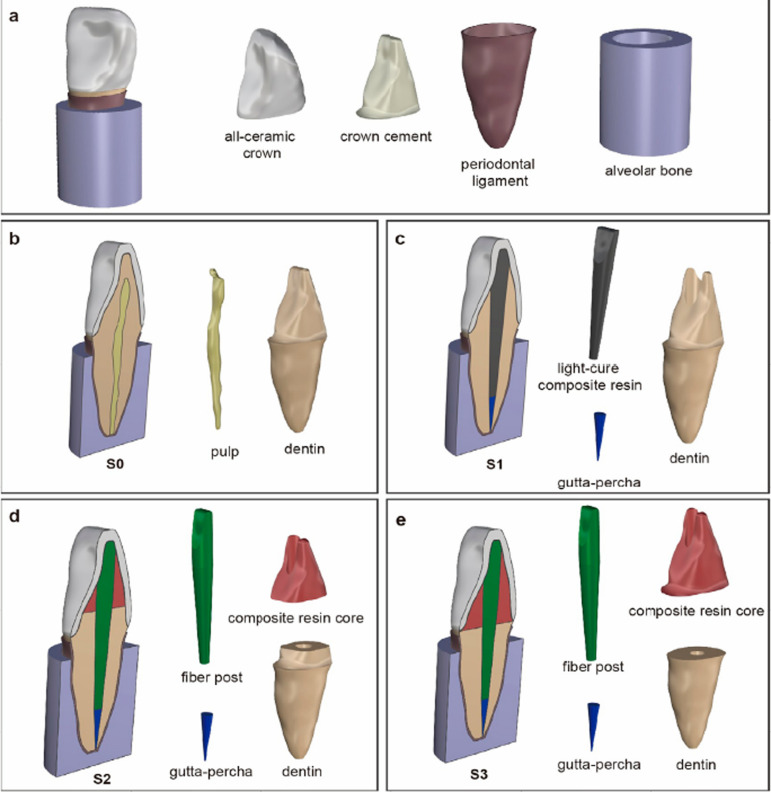
Diagram of models with different constructions, considering D1 models as an example. (a) Depicts the same components of all models: all-ceramic crown, crown cement, periodontal ligament, and alveolar bone. Subsequent panels illustrate variations in construction: (b) S0, (c) S1, (d) S2, and (e) S3.

The groups were further subdivided based on tooth construction. All models were prepared for an all-ceramic crown by removing 1 mm of the incisal edge and preparing a 0.8 mm wide rounded shoulder at the gingival margin, following the minimum clinical recommendations.^12^ The luting cement layer thickness between the prepared tooth and the all-ceramic crown was 0.1 mm.^18^ For S0 models, the pulp was established previously. For S1 models, a root canal was prepared and filled with composite resin and gutta-percha. For S2 and S3 models, the fiber post and resin core were stimulated. Table 1 and Figure 2 present the specific description and diagram of constructions for each model, respectively.

**Table 1:** Description of four different constructions of tooth models.

Model	Description
S0	Vital pulp tooth with all-ceramic crown
S1	ET tooth with all-ceramic crown
S2	ET tooth with all-ceramic crown, with 2-mm ferrule, restored with fiber post (15 mm length, 0.1 taper) and composite resin core
S3	ET tooth with all-ceramic crown, without ferrule, restored with fiber post (15 mm length, 0.1 taper) and composite resin core

### Mesh generation

**Figure 3:**
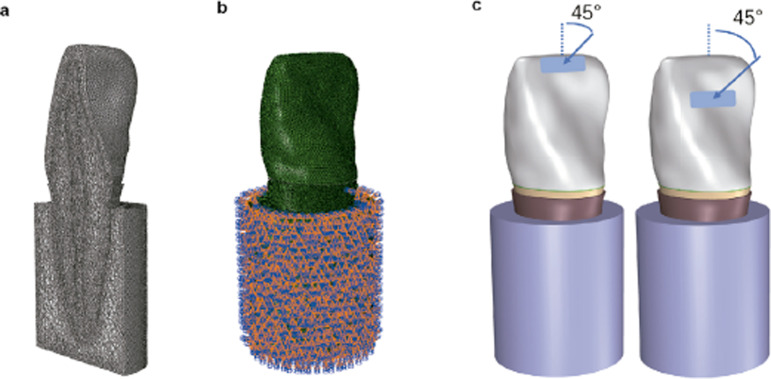
Finite element analysis process. (a) Mesh generation, considering Model (D1, S0) as an example. (b) Boundary condition. (c) Load application.

The 12 geometric models were imported into the HyperMesh software (version 14.0; Altair Engineering, USA), and meshing for each model was performed using a linear tetrahedron (C3D10), as shown in Figure 3a. Table 2 shows the elements and nodes used in each model.

**Table 2:** Information of nodes and elements in the finite element models.

Models	Nodes	Elements
Model (D1, S0)	266 928	1 261 017
Model (D1, S1)	256 467	1 202 516
Model (D1, S2)	240 802	1 125 035
Model (D1, S3)	229 654	1 065 481
Model (D2, S0)	256 925	1 214 437
Model (D2, S1)	246 666	1 156 883
Model (D2, S2)	231 035	1 078 591
Model (D2, S3)	219 821	1 019 102
Model (D3, S0)	246 850	1 166 802
Model (D3, S1)	234 538	1 099 086
Model (D3, S2)	219 487	1 023 887
Model (D3, S3)	211 874	982 109

### boundary condition and load application

The materials used were homogenous, isotropic, linearly elastic, and flawless. The 12 models were fixed in nodes on the outer surface of the alveolar bone (Figure 3b). In addition, the “Tie Contact” condition was applied between each part.

To simulate the cutting function, a static load of 100 N was applied to a 5 mm^2^ area on the incisal edge of the crown at a 45° oblique angle to the longitudinal axis of the teeth (Figure 3c). To simulate the occlusal relationship, the same load was applied to a 5 mm^2^ area on the palatal surface of the crown close to the incisal edge at a 45° oblique angle to the longitudinal axis of the teeth (Figure 3c).

### Material properties

The all-ceramic crown modeled for analysis was a Lithium disilicate crown. The crown cement was composite resin cement. The fiber post modeled was a glass fiber-reinforced post. The core material modeled was composite resin. Table 3 summarizes the values for elasticity modulus and Poisson’s ratio of all materials. The materials used in this study model differed in tensile and compressive strength, but they were isotropic, homogenous, and linearly elastic.^18^

**Table 3:** Mechanical characteristics of the materials.

Materials	Modulus of elasticity (MPa)	Poisson's ratio	References
all-ceramic crown	70 000	0.30	19, 20
dentin	18 600	0.31	21
pulp	2	0.45	21
periodontal ligament	68.9	0.45	22
alveolar bone	13 500	0.31	23
crown cement	8 200	0.30	19
fiber post	23 600	0.32	22
composite resin core	1 2000	0.30	22
light-curing composite resin	8 300	0.28	24
gutta-percha	0.69	0.45	21

### Analysis mode

The FEM results are presented as stresses distributed in the investigated structures. These stresses may occur as tensile, compressive, shear, or a stress combination known as equivalent Von Mises stresses.^22^ Von Mises stresses depend on the entire stress field and are widely used as indicators of possible damage occurrence.^22^ Thus, Von Mises stresses were chosen for the presentation of the maximum stress and stress distribution of models (dentin, post, and core).

## Results

**Figure 4:**
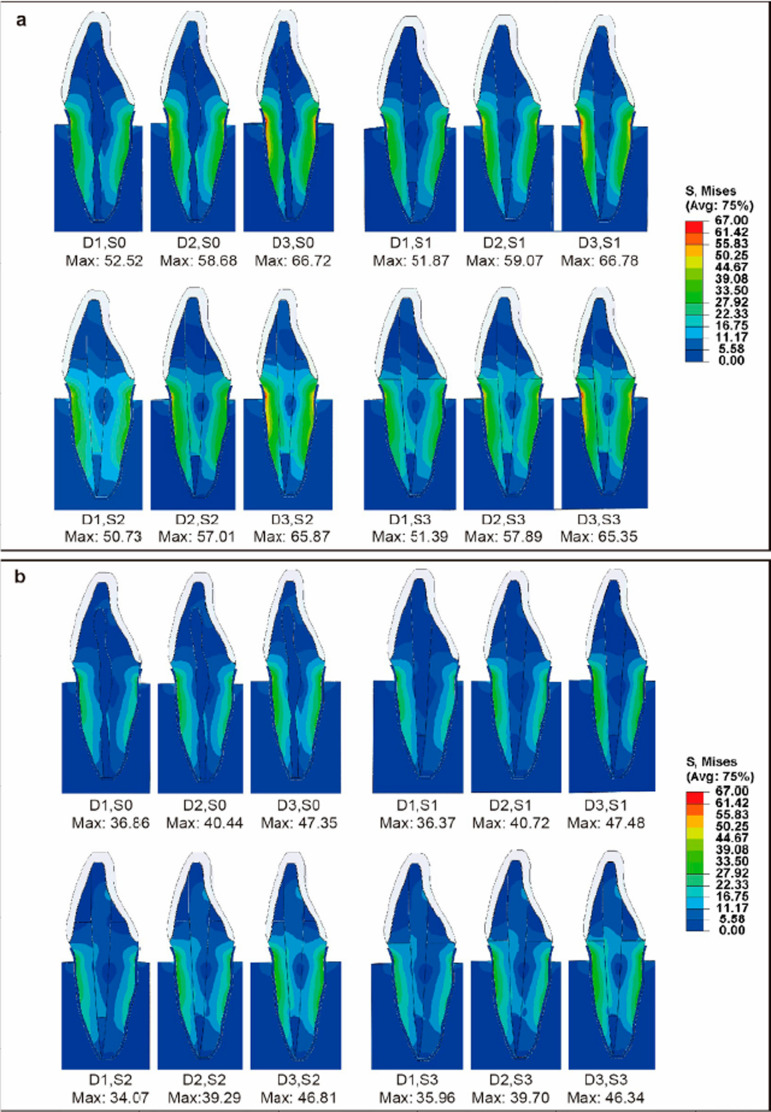
Distribution of Von Mises stresses (MPa) in the labiolingual section of 12 models under a static 100 N load. (a) The loading location was the incisal edge. (b) The loading location was the palatal surface.

Figure 4 shows the stress distribution diagrams for all 12 models. A similar stress distribution pattern was observed across all 12 models under both loading locations, with the highest stress regions concentrated in the labial cervical 1/3 of each root.

**Figure 5:**
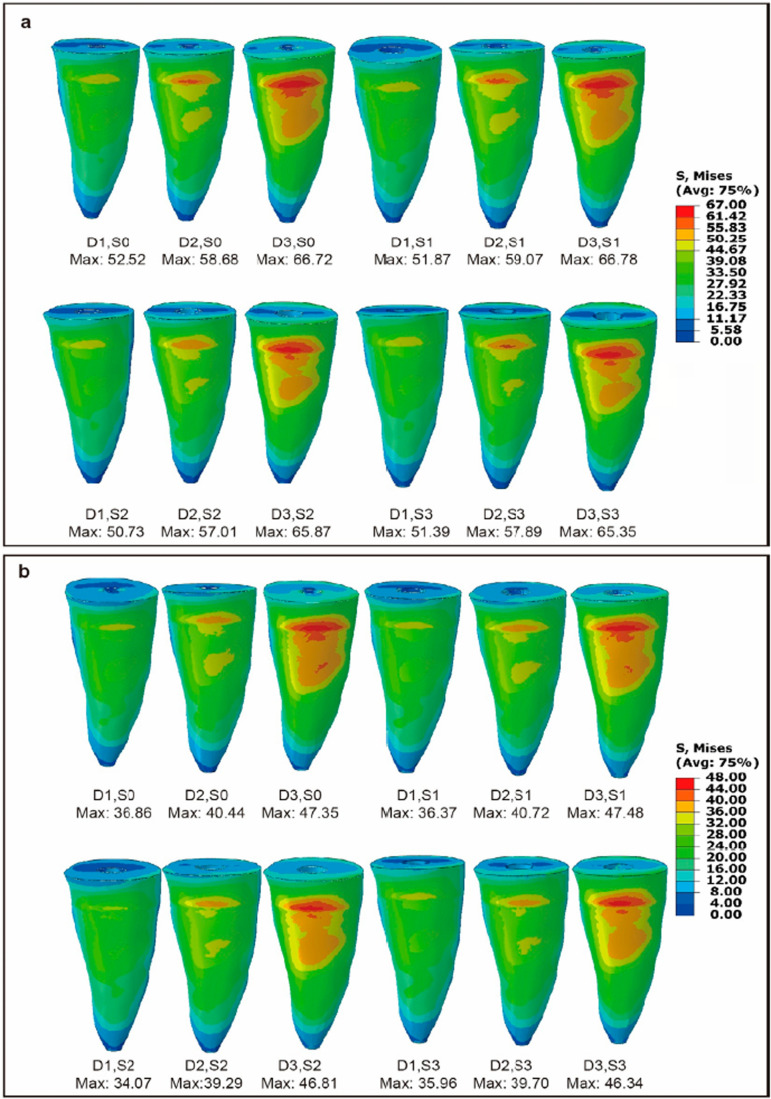
Distribution of Von Mises stresses (MPa) in the root of 12 models, at the labial view. (a) The loading location was the incisal edge. (b) The loading location was the palatal surface.

**Figure 6:**
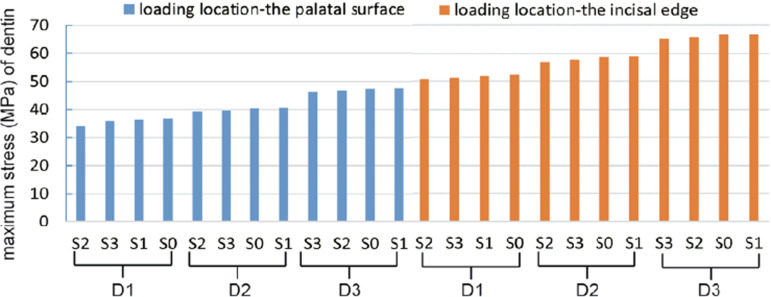
Maximum stresses of residual tooth tissue in each model under a static 100 N load arranged from small to large.

Figure 5 shows the stress distribution diagrams of roots. Meanwhile, Figure 6 presents the maximum stresses, arranged from small to large. It illustrates that the maximum stress on the residual tooth tissue was influenced by the loading location, a factor more impactful than the labiolingual diameter or tooth construction. The highest maximum stress (66.78 MPa) among models occurred on Model (D3, S1), with the incisal edge as loading location. Meanwhile, the lowest maximum stress (34.07 MPa) occurred on Model (D1, S2) on the palatal surface. Both labiolingual diameter and construction affected the maximum stresses of the residual tooth tissue, with the labiolingual diameter demonstrating a more pronounced impact than that of the construction. As the labiolingual diameter decreased, the maximum stress increased. When the loading location was on the incisal edge, the maximum stress was as follows: D1 models (50.73~52.52 MPa) < D2 models (57.01~59.07 MPa) < D3 models (65.35~66.78 MPa); when the loading location was on the palatal surface, a similar conclusion was found: D1 models (34.07~36.86 MPa) < D2 models (39.29~40.72 MPa) < D3 models (46.34~47.48 MPa).

**Figure 7:**
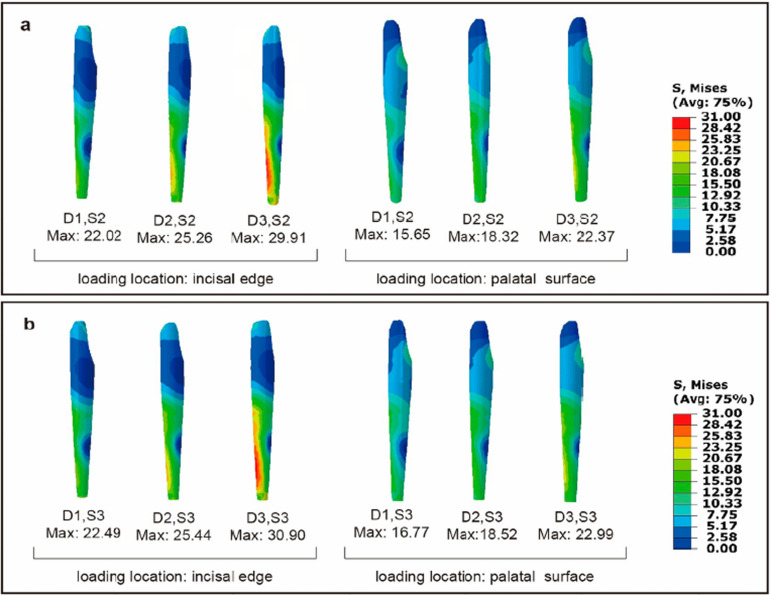
Distribution of Von Mises stresses (MPa) in the post of 12 models, at the labial view. (a) The post of S2 models. (b) The post of S3 models.

**Figure 8:**
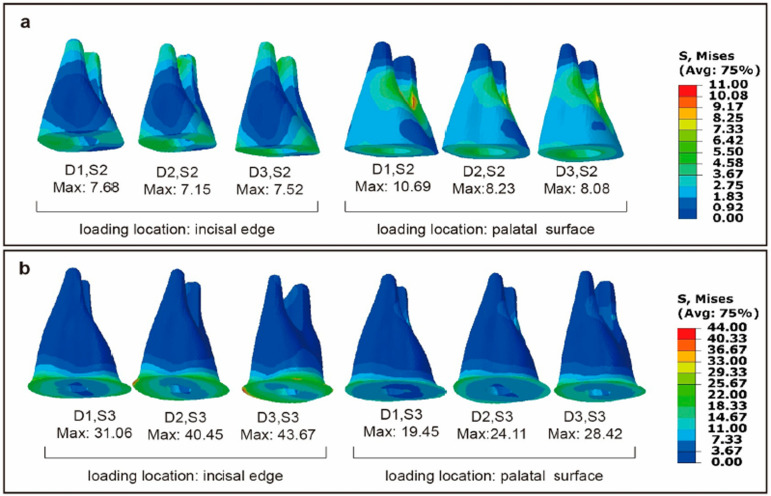
Distribution of Von Mises stresses (MPa) in the core of 12 models, at the labial view. (a) The core of S2 models (b) The core of S3 models.

With the same loading location and labiolingual diameter, little differences were found on stress distribution and maximum stress of residual tooth tissue between the S2 and S3 models, which was less than that of the S0 and S1 models, as shown in Figure 5 and Figure 6. Similarly, also with the same loading location and same labiolingual diameter, the stress distribution and maximum stress of posts in the S2 and S3 models showed little difference, as illustrated in Figure 7. However, with the same loading location and same labiolingual diameter, the maximum stress of cores in the S2 models (7.15~10.69 MPa) were dramatically low compared to the S3 models (19.45~43.67 MPa); moreover, the stress distribution of the core in the S2 models was more uniform than that of S3 models, as illustrated in Figure 8.

## Discussion

In this study, regardless of the loading location, the stress concentration for all models occurred in the labial cervical 1/3 of each root. This is consistent with previous literature indicating that, unlike posterior teeth that experience compressive forces during chewing, the maxillary central incisors undergo high shear forces during cutting function or occlusion, making the root cervix a vulnerable area for fracture failures.^1,25,26^ Moreover, the loading location had a bigger effect on the maximum stresses throughout the tooth than the other two factors (the labiolingual diameter and tooth construction). Shifting the loading location from the palatal surface to the incisal edge resulted in a rapid increase in maximum stress. Therefore, careful consideration of the appropriate repair method is essential for anterior teeth subjected to excessive cutting function.

Additionally, variations in labiolingual diameters and tooth constructions were found to impact the maximum stresses. Notably, the influence of labiolingual diameter was more important than that of the construction. Regardless of the tooth construction (vital pulp teeth, ET teeth, or ET teeth with post-core restoration), the maximum stress throughout the teeth increased as the labiolingual diameter decreased. This indicates that a decreased labiolingual diameter could affect ET anterior teeth biomechanics, and small labiolingual diameters could lead to a high risk of tooth fracture. However, further exploration is required to determine the significance of the labiolingual diameter as a potential factor leading to restoration failure.

The amount of residual tooth tissue, recognized as the most critical factor influencing restoration failure, is directly associated with fracture resistance and longevity.^3,25,27,28^ In this study, under the same labiolingual diameter and loading location, little difference was observed in the maximum stresses between the S0 and S1 models. This implies that root canal therapy had a minimal impact on the maximum stress of the tooth when sufficient residual coronal tooth tissue was present and restored with an all-ceramic crown. A few studies have similarly concluded that post is not necessary for an anterior tooth with normal size and minimal loss of tooth structure (such as proximal Class III cavities) but with pulpal or periapical lesions that requires the root canal treatment.^1,5^

However, restoration of ET teeth with relatively small labiolingual diameters and limited residual tooth tissue on the coronal side requires further consideration. In clinical practice, when the peri cervical region experiences diminished dentin availability, the risk of crown or root fracture increases.^3,29,30^ In this study, teeth with reduced residual tooth tissue were observed to be more susceptible to deformation and failure under both axial and lateral stresses. Fiber posts and composite resin cores were used in this study, in which a large loss of tooth structure occurred. The ferrule, being one of the methods for evaluating the amount of residual tooth tissue, has been recognized in numerous studies for its impact on fracture resistance.^3,10,22,25,28,31^ In this study, the presence of a ferrule did not improve the maximum stress of the residual tooth tissue, but dramatically reduced that of the composite resin core, and a homogeneous stress distribution was observed (Figure 8). Thus, the presence of the ferrule remarkably reduced the maximum stress difference between the composite resin core and fiber post. Materials with a higher elastic modulus concentrate more stresses^18^ and may transmit undamped stresses at the post and core bonded interface. It has been widely proven that failure in the bonding interface between the fiber post and composite resin core is one of the preliminary failures due to differences in the elastic modulus.^5,11,25^ Based on this study, it can be inferred that the present of a 2-mm ferrule could improve the bonding effect on post and core, as well as further improve the service life of restoration. Besides, the placement of posts did not largely improve the maximum stress of the ET teeth in this study. This aligns with the current consensus^5,6^ that posts are widely used in teeth with insufficient coronal structures to provide retention for composite resin cores and crowns, rather than increasing the flexural strength.

This study presents a few limitations. The model assumed homogeneous, isotropic structures with linear elasticity. However, the properties of the materials modelled in this study, particularly those of living tissues, differed. For instance, it was well-described that periodontium is transversely isotropic and inhomogeneous.^22,32^ Thus, the inherent limitations of this study should be considered. Clinical experience indicates that most fractures in prosthodontic restorations occur after several years.^22^ Generally, such failures are unrelated to episodes of acute overload but result from fatigue failure.^22^ The absence of fatigue loading is another limitation of the study.

In summary, the reduction of labiolingual diameter induced by clinical excessive preparation, aging, and ethnicity could increase the failure risk of crown restorations. Therefore, crown restorations should be used with caution for anterior teeth with smaller labiolingual diameters in clinical practice. In addition, with the development of dental materials, we must seek better anterior tooth ceramics and composite materials that can combine aesthetics and biomechanics.

## Conclusions

The reduction in the labiolingual diameter exerts a greater impact on the biomechanical behavior of the ET anterior teeth with crown restoration than the construction. For anterior crown restorations supported by a fiber post and composite resin core, the ferrule can reduce the maximum stress of the composite resin core and maintain the uniformity of the stress distribution.
